# Spinal CCL2 Promotes Pain Sensitization by Rapid Enhancement of NMDA-Induced Currents Through the ERK-GluN2B Pathway in Mouse Lamina II Neurons

**DOI:** 10.1007/s12264-020-00557-9

**Published:** 2020-08-18

**Authors:** Hui Zhang, Sui-Bin Ma, Yong-Jing Gao, Jun-Ling Xing, Hang Xian, Zhen-Zhen Li, Shu-Ning Shen, Sheng-Xi Wu, Ceng Luo, Rou-Gang Xie

**Affiliations:** 1 Department of Neurobiology, Fourth Military Medical University, Xi’an, 710032 China; 2 Department of Health Statistics, Fourth Military Medical University, Xi’an, 710032 China; 3 Pain Research Laboratory, Institute of Nautical Medicine, Jiangsu Key laboratory of Neuroregeneration, Nantong University, Nantong, 226001 China; 4 Department of Radiation Biology, Faculty of Preventive Medicine, Fourth Military Medical University, Xi’an, 710032 China; 5 Department of Orthopedics, Xijing Hospital, Fourth Military Medical University, Xi’an, 710032 China; 6 Department of Stomatology, No. 984 Hospital of the People’s Liberation Army, Beijing, 100094 China

**Keywords:** C-C motif chemokine ligand 2, Monocyte chemoattractant protein 1, Neuron-glial interaction, Extracellular signal-regulated kinase

## Abstract

Previous studies have shown that CCL2 (C–C motif chemokine ligand 2) induces chronic pain, but the exact mechanisms are still unknown. Here, we established models to explore the potential mechanisms. Behavioral experiments revealed that an antagonist of extracellular signal-regulated kinase (ERK) inhibited not only CCL2-induced inflammatory pain, but also pain responses induced by complete Freund’s adjuvant. We posed the question of the intracellular signaling cascade involved. Subsequent experiments showed that CCL2 up-regulated the expression of phosphorylated ERK (pERK) and N-methyl D-aspartate receptor [NMDAR] subtype 2B (GluN2B); meanwhile, antagonists of CCR2 and ERK effectively reversed these phenomena. Whole-cell patch-clamp recordings revealed that CCL2 enhanced the NMDAR-induced currents *via* activating the pERK pathway, which was blocked by antagonists of GluN2B and ERK. In summary, we demonstrate that CCL2 directly interacts with CCR2 to enhance NMDAR-induced currents, eventually leading to inflammatory pain mainly through the CCL2–CCR2–pERK–GluN2B pathway.

## Introduction

Most known inflammatory mediators cause pain by binding to nociceptors located in the peripheral nervous system [1–3]. Recently, it has been recognized that neuroinflammation plays a key role in the pathogenesis of neuropathic and inflammatory pain [4, 5]. Increasing evidence suggests that microglia play important roles in the development and progression of chronic pain [6, 7]. Microglia can be rapidly activated by small pathological changes in the central nervous system.

Chemokines are a family of secreted small molecules that regulate inflammatory responses in the dorsal root ganglia, spinal cord, and brain [5, 8–10]. Increasing evidence suggests that chemokines are associated with chronic pain after nerve injury [11, 12] and chronic itch [13]. C-C motif chemokine ligand 2 (CCL2; also known as monocyte chemotactic protein 1) specifically recruits monocytes to the sites of inflammation, infection, or trauma. Evidences have suggested that CCL2 and CCR2 (CC receptor 2) are involved in neuropathic pain [14, 15]. Intrathecal injection of CCL2 causes inflammatory hyperalgesia [16]. A significant reduction in mechanical allodynia occurs after partial ligation of the sciatic nerve in mice lacking CCR2 [17, 18]. However, the cellular mechanism of CCL2-induced pain sensitization is entirely not clear.

Our previous research showed that CCL2 directly regulates the synaptic plasticity of excitatory neurons expressing CCR2 in spinal cord outer lamina II (IIo), and this is the basis of the central sensitization in chronic pain [16]. However, some issues still need to be further clarified, such as: the downstream pathway of CCL2/CCR2; the molecules through which CCL2/CCR2 regulates NMDARs; and the selective specific signaling pathway through which CCL2/CCR2 regulates NMDARs. Using patch-clamp recordings together with biochemical and behavioral assays, we demonstrated that upon peripheral inflammation or injury, released CCL2 directly acts on CCR2 in superficial dorsal horn neurons, leading to activation of ERK signals and GluN2B upregulation, which in turn contribute to the development of inflammatory pain.

## Methods

### Animals and Pain Models

C57Bl/6 background wild-type (WT) mice were purchased and bred in the Animal Facility of the Fourth Military Medical University. Young C57Bl/6 mice (4–6 weeks old) were used for electrophysiological studies in spinal cord slices, and adult CD1 and C57Bl/6 mice (male, 8–10 weeks old) were used for behavioral and pharmacological experiments. All the animal procedures were approved by the Animal Care Committee of the Fourth Military Medical University. To produce persistent inflammatory pain, complete Freund’s adjuvant (CFA, 20 μL, 1 mg/mL, Sigma, St Louis, MO, USA) was injected into the plantar surface of a hind paw.

### Behavioral Analysis

Mice were habituated to the testing environment for at least 2 days before baseline testing. Thermal hyperalgesia and mechanical allodynia were tested as previously described [16, 19]. The experimenters were blinded to treatments.

### Western Blot

Protein samples were prepared in the same way as for ELISA analysis; they were separated on SDS-PAGE gels and transferred to nitrocellulose blots. The blots were blocked and incubated overnight at 4°C with antibodies against ERK, GluN1, and GluN2B (1:50, rabbit; Boster, Fremont, CA), GluN2A (1:500, rabbit, Abcam, Cambridge, MA), and CCL2 and CCR2 (1:500, rabbit; Novus Biologicals, Centennial, CO).

### Spinal Cord Slice Preparation and Patch-Clamp Recordings

As we previously reported [16], the Krebs’ solution contained (in mmol/L): 117 NaCl, 3.6 KCl, 2.5 CaCl_2_, 1.2 MgCl_2_, 1.2 NaH_2_PO_4_, 25 NaHCO_3_, and 11 glucose. After establishing the whole-cell configuration, neurons were held at –70 mV to record evoked excitatory postsynaptic currents (eEPSCs) by stimulating the dorsal root entry zone *via* a concentric bipolar electrode using an isolated current stimulator [19]. The internal solution contained (in mmol/L): 110 Cs_2_SO_4_, 2 KCl, 0.1 CaCl_2_, 2 MgCl_2_, 1.1 EGTA, 10 HEPES, 5 ATP-Mg. QX-314 (5 mmol/L) was added to the pipette solution to prevent the discharge of action potentials. To induce spinal long-term potentiation (LTP), low-frequency conditioning stimulation (240 pulses at 2 Hz) was applied to the dorsal root with the same intensity as test stimulation. Signals were filtered at 2 kHz and digitized at 5 kHz. Data were stored and analyzed on a personal computer using pCLAMP10 software (Molecular Devices, San Jose, CA).

### Reverse-Transcription PCR (RT- PCR)

Total RNA was extracted using TRIzol reagent and was reverse transcribed using an oligo (dT) primer. qPCR analysis was performed with the Real-Time Detection System by SYBR green I dye detection (Takara, Shiga, Kusatsu, Japan).

### Immunofluorescence Labelling

Immunohistochemistry was performed according to standard protocols, and the following primary antibodies were used: pERK (rabbit, 1: 200, Cell Signaling, Danvers, MA, USA), Alexa Fluor® 488 (donkey anti-rabbit IgG, 1:1000, Molecular Probes, Waltham, MA), CGRP antibody (goat, 1:300, Abcam, Cambridge, MA), CCR2 antibody (rabbit, 1:300, Novus Biologicals, Centennial, CO, USA), Alexa Fluor® 594 (donkey anti-rabbit IgG, 1:1000, Abcam), pERK antibody (mouse, 1: 300, Abcam), and Alexa Fluor® 594 (donkey anti-mouse IgG, 1:1000, Abcam).

### Drugs and Administration

The mitogen-activated protein kinase (MEK1) inhibitor PD98059 (Cell Signaling Technology, EMD Millipore, Billerica, MA, USA), the CCR2 antagonist, RS504393 (Tocris Bioscience, Bristol, UK), CCL2 (R & D Systems, Minneapolis, MN, USA), and the selective blocker of the NMDAR GluN2B subunit, Ifenprodil, (Sigma, New York, NY) were used in this study. For intrathecal injection, under brief anesthesia with isoflurane a lumbar puncture was made at L5–L6 with a 30-gauge needle as previously described [20].

### Statistical Analysis

Differences between groups were compared using 1-way ANOVA or 2-way repeated measures ANOVA followed by Bonferroni’s test or by Student’s *t* test (2-tailed) if only 2 groups were compared. The criterion for a statistically significant difference was *P* < 0.05.

## Results

### ERK Mediates CCL2- and CFA-Induced Mechanical Hyperalgesia

**Fig. 1:**
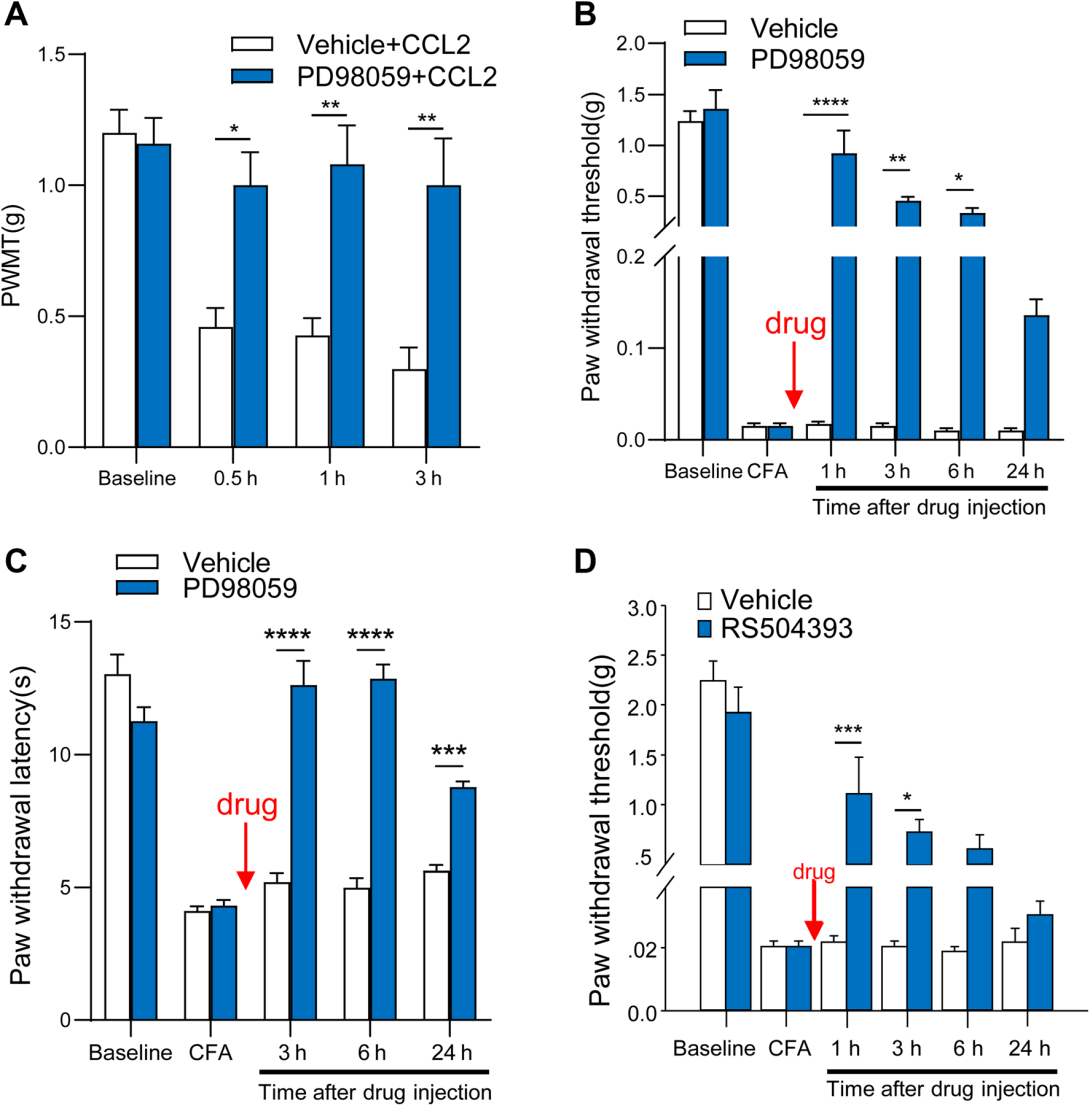
Inhibition of CCL2- and CFA-induced mechanical hyperalgesia by intrathecal injection of RS504393 or PD98059. **A** Prevention of CCL2 (100 ng, i.t.)-induced mechanical hyperalgesia by PD98059 (1 g, i.t.). **B** Reversal of CFA-induced mechanical hyperalgesia by PD98059 (1 μg, i.t.) given 1 day after CFA injection. **C** Reversal of CFA-induced thermal hyperalgesia by PD98059 (1 μg, i.t.) given 1 day after CFA injection. **D** Reversal of CFA-induced mechanical hyperalgesia by RS504393 (20 μg, i.t.) given 1 day after CFA injection. *P < 0.05, **P < 0.01, ***P < 0.001, ****P < 0.0001, *n* = 5–6.

To further investigate the mechanism of spinal sensitization induced by CCL2, we tested the behavioral involvement of spinal ERK in CCL2- and CFA-induced hyperalgesia. Consistent with our previous reports [16], intrathecal CCL2 (100 ng) induced rapid mechanical hyperalgesia, characterized by a prominent drop in mechanical threshold, while intrathecal administration of an ERK inhibitor, PD98059 (1 μg), potently abolished this hyperalgesia (Fig. 1A). Intraplantar CFA injection induced marked mechanical hyperalgesia (Fig. 1B) and thermal hyperalgesia (Fig. 1C) on day 1 in C57Bl/6 mice. It is worth noting that the mechanical hyperalgesia was completely reversed by PD98059 (1 μg) at 1 h after intrathecal injection (Fig. 1B) and the thermal hyperalgesia was completely reversed by PD98059 (1 μg) at 3 h after intrathecal injection (Fig. 1C). This reversal was transient and was recovered within 24 h after antagonist treatment (Fig. 1B). Furthermore, intraplantar CFA injection induced significant mechanical hyperalgesia on day 1 in CD1 mice as well (Fig. 1C), and this was remarkably attenuated by RS504393 (20 μg, i.t.), a CCR2 antagonist, at 1 h after injection, and recovered 6 h after antagonist treatment (Fig. 1D), consistent with the results of our previous testing of thermal pain sensitivity [16]. These results indicate that CCL2- and inflammation-mediated mechanical hyperalgesia require the involvement of ERK.

### CFA Induces Upregulation of Spinal CCL2, CCR2, and pERK

**Fig. 2:**
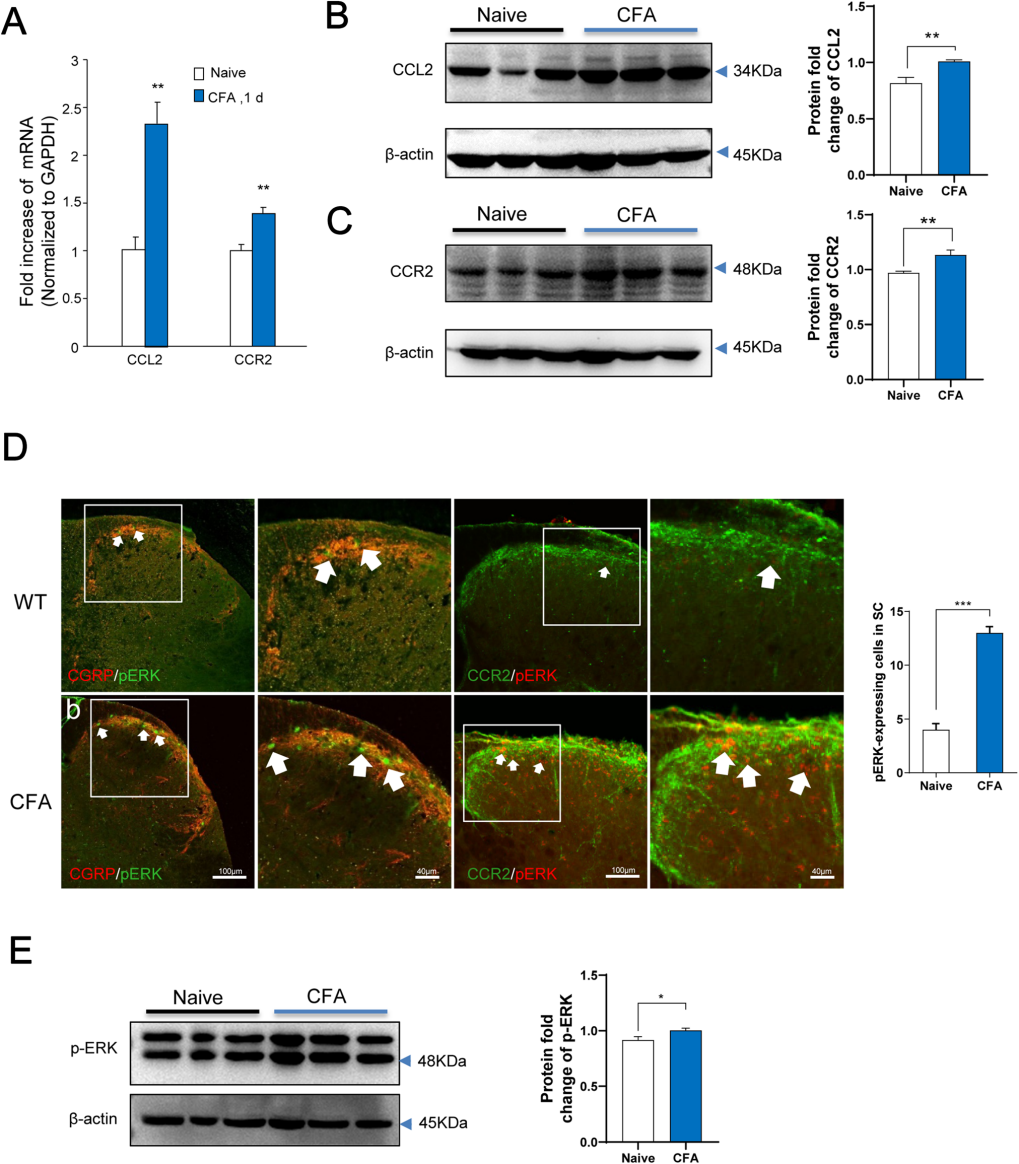
CFA induces upregulation of CCL2, CCR2, and ERK activation in the spinal dorsal horn. **A** Real-time PCR reveals distinct regulation of CCL2 and CCR2 mRNA expression in the spinal cord following inflammation. Both CCL2 and CCR2 are upregulated after inflammatory pain. CFA induces a transient increase in CCL2 and CCR2 mRNA levels on day 1 (**P < 0.01, *n* = 6–7). **B**, **C** Western blots showing that CFA leads to a significant increase in CCL2 and CCR2 expression. **D** Immunofluorescence staining showing an increase in pERK-immunoreactive (pERK-ir) cells in CCR2-expressing neurons in the ipsilateral (injured) dorsal horn at 24 h after CFA (right panels, enlargement of the white frames; arrows, pERK-ir cells in the superficial dorsal horn). **E** Western blot showing that CFA results in a significant increase in pERK expression.

We further applied real-time PCR to study CCL2 and CCR2 mRNA expression in the spinal cord after inflammation, and both were upregulated. Also, a dramatic increase of the CCL2 and CCR2 mRNA levels was detected in the spinal dorsal horn on day 1 after CFA injection (Fig. 2A). Western blot analysis showed that CFA inflammation led to a significant increase in CCL2 expression as well as CCR2 expression (Fig. 2B, C). CCL2 localization in the spinal cord has been clearly reported in previous studies. For example, CCL2 is mainly localized in lamina II [11, 21], and is also found in some central terminals of primary afferents that express CGRP-IR (calcitonin gene-related peptide immunoreactive) in the superficial spinal cord [15]. Previous immunohistochemical studies have shown CCR2 expression in spinal microglial cells [18], astrocytes [22],and neurons [15]. CCL2 is mainly expressed on isolectin B4-positive and CGRP-positive neurons in dorsal root ganglia [23]. We further assessed the expression of pERK in CGRP-positive primary afferent terminals. Immunofluorescence staining revealed that CFA inflammation caused marked pERK1/2 induction in the dorsal horn, especially in the superficial dorsal horn where nociceptive primary afferents marked by CGRP mainly terminate (Fig. 2D). Also double-staining of CCR2 and pERK1/2 showed a marked increase in co-expressing neurons (Fig. 2D). Meanwhile, western blots showed that CFA resulted in a significant increase in pERK expression (Fig. 2E). These results suggest that peripheral inflammation activates CCL2/CCR2 and the ERK signaling pathway in the superficial dorsal horn.

### CCL2 Enhances NMDA Currents in Spinal Dorsal Horn Neurons via ERK Activation

**Fig. 3:**
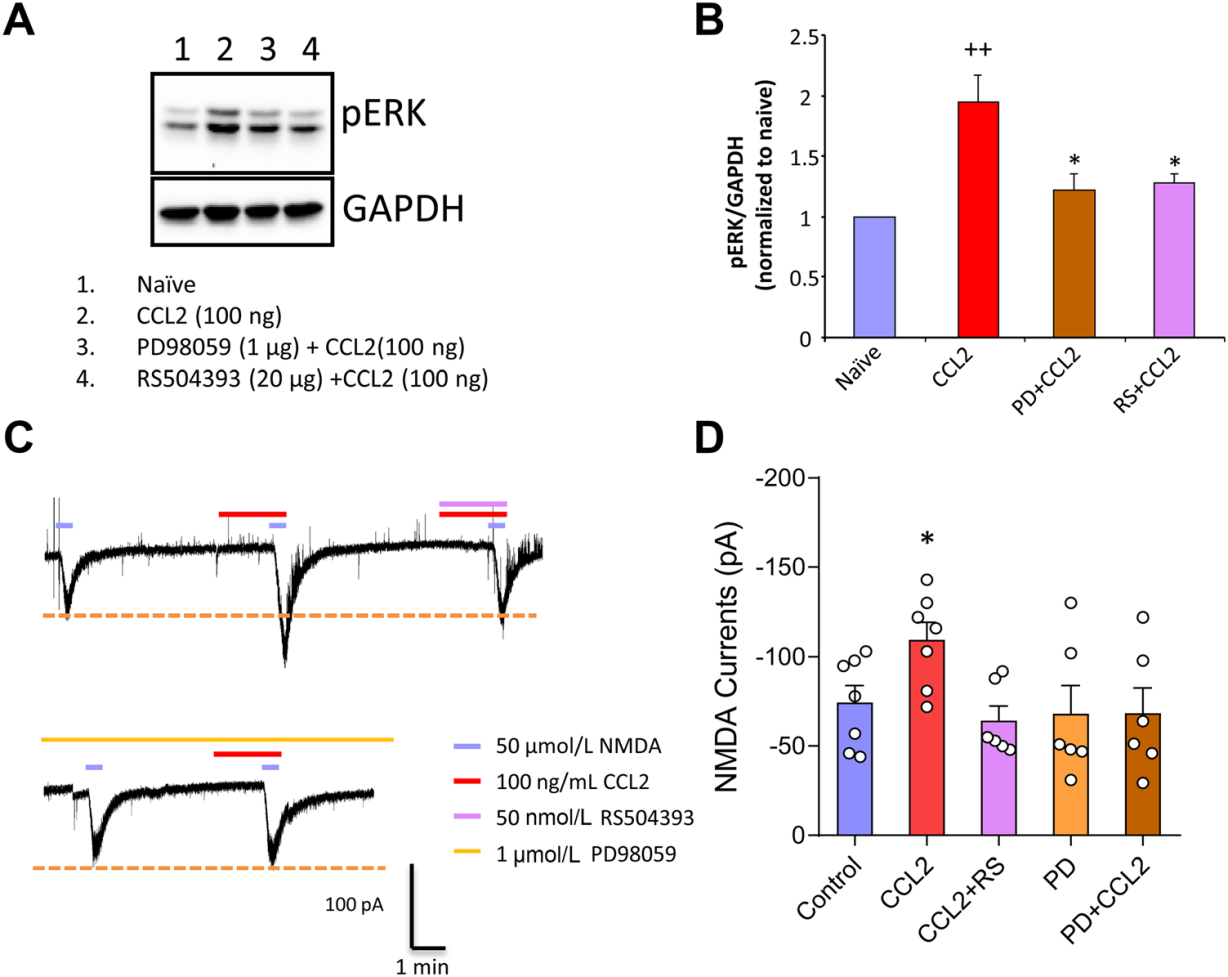
CCL2 enhances NMDA currents *via* ERK activation. **A, B** Western blots showing CCL2 directly increases neuronal pERK levels, and this effect is blocked by intracellular PD98059 (pERK blocker) and extracellular RS504393 (CCR2 inhibitor) (^++^*P* < 0.01 *vs* Naïve, *P < 0.05 *vs* CCL2, *n* = 6–7). **C, D** Superfusion of NMDA (50 μmol) at a holding potential of –40 mV, induces a robust inward current in WT mice. Superfusion of CCL2 at 100 ng/mL increases NMDA-induced currents (*P < 0.05, *n* = 6–7), and this is abolished by the antagonist RS504393. The ERK blocker PD98059 in the pipette solution abolishes the enhancement effect of CCL2 on NMDA-induced currents on lamina IIo neurons.

Previous studies have shown that CCL2 enhances NMDA currents in spinal dorsal horn neurons [16], so we next sought to identify which pathway mediates this enhancement. Western blotting analysis was performed in the spinal dorsal horn from naïve mice and mice with intrathecal delivery of CCL2 in the absence and presence of PD98059 (1 μg) or RS504393 (20 μg). Compared to naïve mice, intrathecal administration of CCL2 (100 ng) evoked significant upregulation of pERK at 6 h after treatment (Fig. 3A, B). Of note, this exaggerated increase of pERK was significantly depressed by intrathecal delivery of PD98059 and RS504393 (Fig. 3A, B), suggesting the involvement of pERK activation in the CCL2/CCR2 pathway.

In order to confirm the functional role of pERK enhancement, we tested NMDA-induced currents in spinal lamina IIo neurons. As shown in Fig. 3C, CCL2 superfusion caused a marked increase of these currents. Antagonizing CCR2 with RS504393 (50 nmol) significantly suppressed this enhancement, suggesting that CCL2 enhances NMDAR function *via* activation of CCR2. To further determine whether ERK activation links the interaction between CCL2 and NMDARs, we included PD98059 in the pipette solution and found that it completely abolished the enhancement of NMDA currents by CCL2 (Fig. 3C, D). We infer from the above that CCL2 enhances NMDAR function *via* the pERK signaling pathway.

### Endogenous CCL2 and CCR2 are Involved in the Enhancement of NMDA Currents in the Inflammatory State

**Fig. 4:**
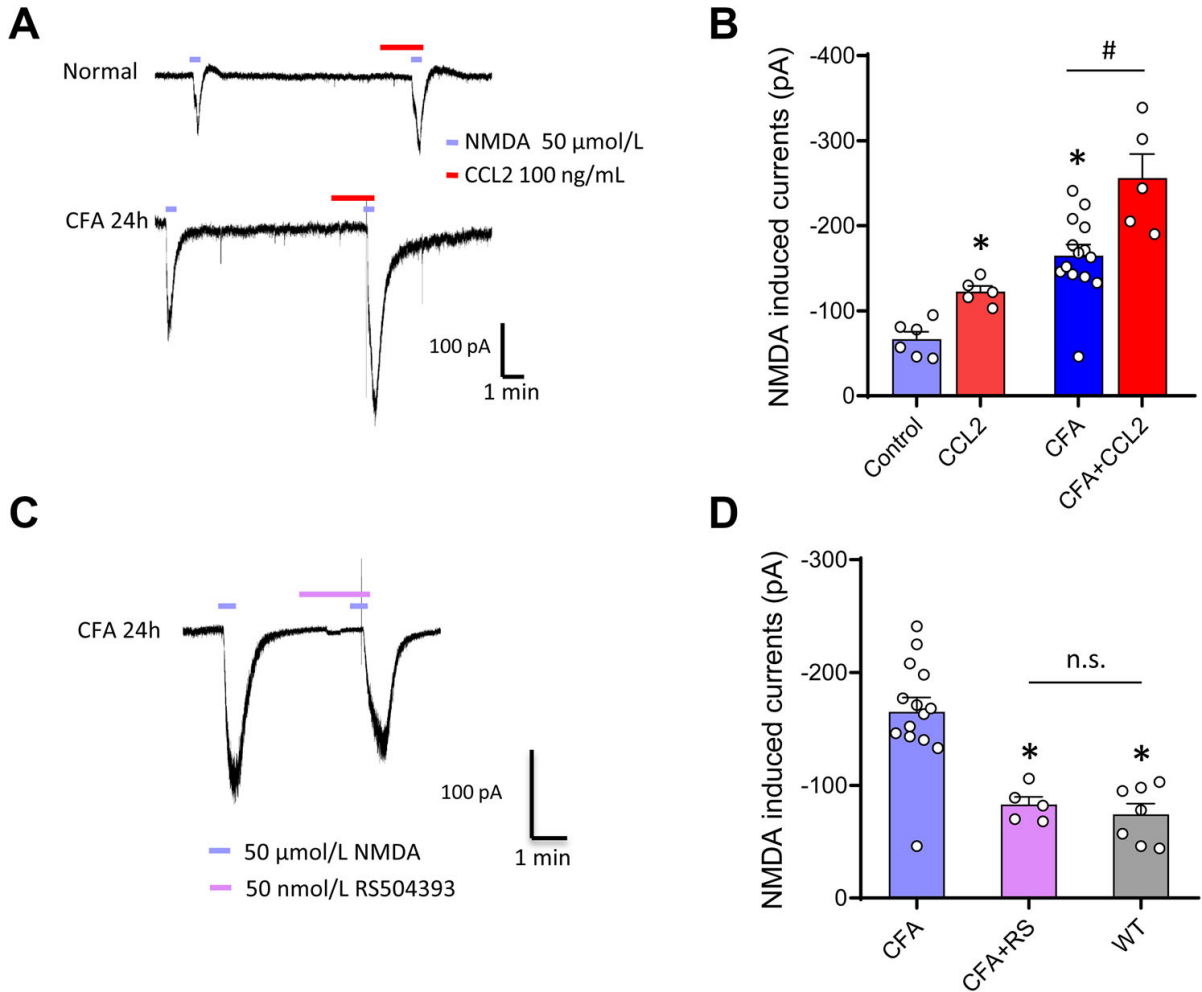
Role of endogenous CCR2 and effects of CCR2 antagonist on NMDA currents after inflammation. **A, B** NMDA-induced currents are enhanced in spinal lamina IIo neurons in both naïve (A, upper panels) and inflammatory (A, lower panels) states. **C, D** Superfusion of the CCR2 antagonist RS504393 eliminates the facilitation of NMDA-induced currents by CFA inflammation in spinal lamina IIo neurons. *P < 0.05, *n* = 5–14.

In addition to the enhancement of NMDA currents by CCL2 in naïve mice, we next addressed whether CCL2 can further increase NMDA currents in inflammatory states. Consistent with previous reports [16], these currents displayed robust upregulation upon CFA inflammation (Fig. 4A, B). Similar to the facilitation of NMDA currents by CCL2 in the naïve state, the increase in currents by inflammation was further enhanced by CCL2 (Fig. 4A, B). Given the fact that peripheral inflammation was able to induce CCL2 expression in the spinal cord, we next asked whether the enhancement of NMDA currents by inflammation is mediated by endogenous CCL2 release. The CFA-induced facilitation of the currents was completely eliminated by blockade of CCR2 with pretreatment of RS504393 in spinal lamina IIo neurons (Fig. 4C, D). These results indicate that the endogenous CCL2/CCR2 signaling cascade contributes to enhancement of NMDAR function after paw inflammation.

### CCL2 Enhances NMDA Currents via GluN2B Activation and a GluN2B Antagonist Attenuates CFA-Induced Pain Hypersensitivity

**Fig. 5:**
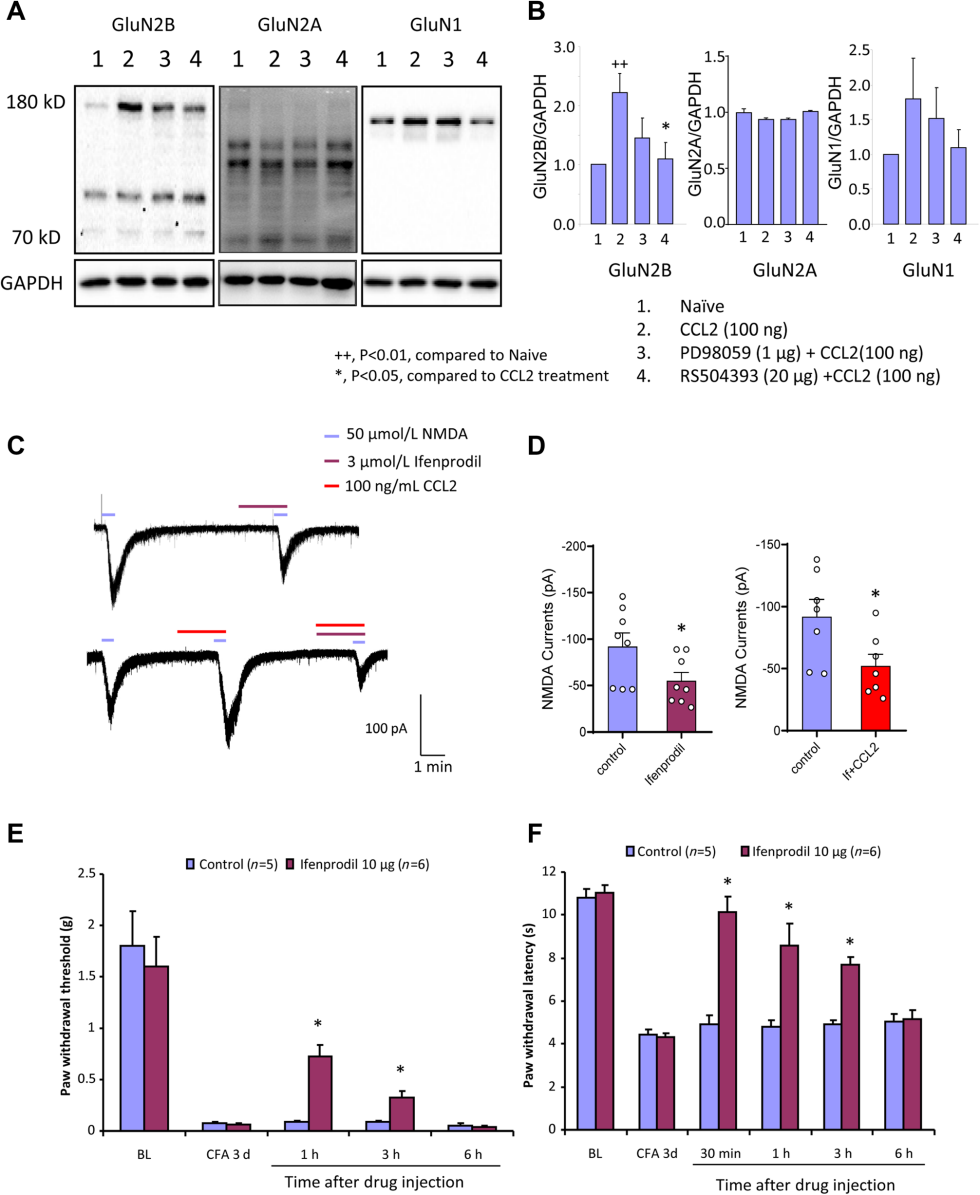
CCL2 enhances NMDA currents *via* GluN2B activation. **A, B** Western blots showing that CCL2 induces a significant increase in GluN2B expression. **C, D** Ifenprodil, a GluN2B blocker, partially inhibits NMDAR-induced currents in lamina IIo neurons and abolishes the CCL2-induced enhancement of these currents. **E, F** Behavioral tests showing that CFA-induced mechanical allodynia and thermal hyperalgesia are reversed by i.p. Ifenprodil. *P < 0.05, ^++^*P* < 0.01, *n* = 6–8.

To further address how CCR2 works on NMDAR function, we assessed the expression of different subtypes of NMDAR (GluN1, GluN2A, and GluN2B subunits) by western blot analysis. Our results demonstrated that CCL2 led to a dramatic increase in GluN2B expression, but no significant changes in GluN2A and GluN1 expression (Fig. 5A, B), and RS504393 partly abolished the facilitation of GluN2B by CCL2. In support of these results, patch-clamp recording from lamina IIo neurons demonstrated that Ifenprodil, a specific blocker of GluN2B, which partially inhibits NMDA currents in these neurons, abolished the facilitation of NMDA currents by CCL2 (Fig. 5C, D). This indicates that CCL2 exerts its major effect on the GluN2B subunit in spinal lamina IIo neurons. Furthermore, we demonstrated that blockade of the GluN2B subunit with Ifenprodil strongly reversed the maintained mechanical allodynia as well as the thermal hyperalgesia induced by CFA inflammation (Fig. 5E, F).

### Enhancement of NMDA Currents by CCL2/CCR2 is Mediated *via* the Cox-2 Pathway

**Fig. 6:**
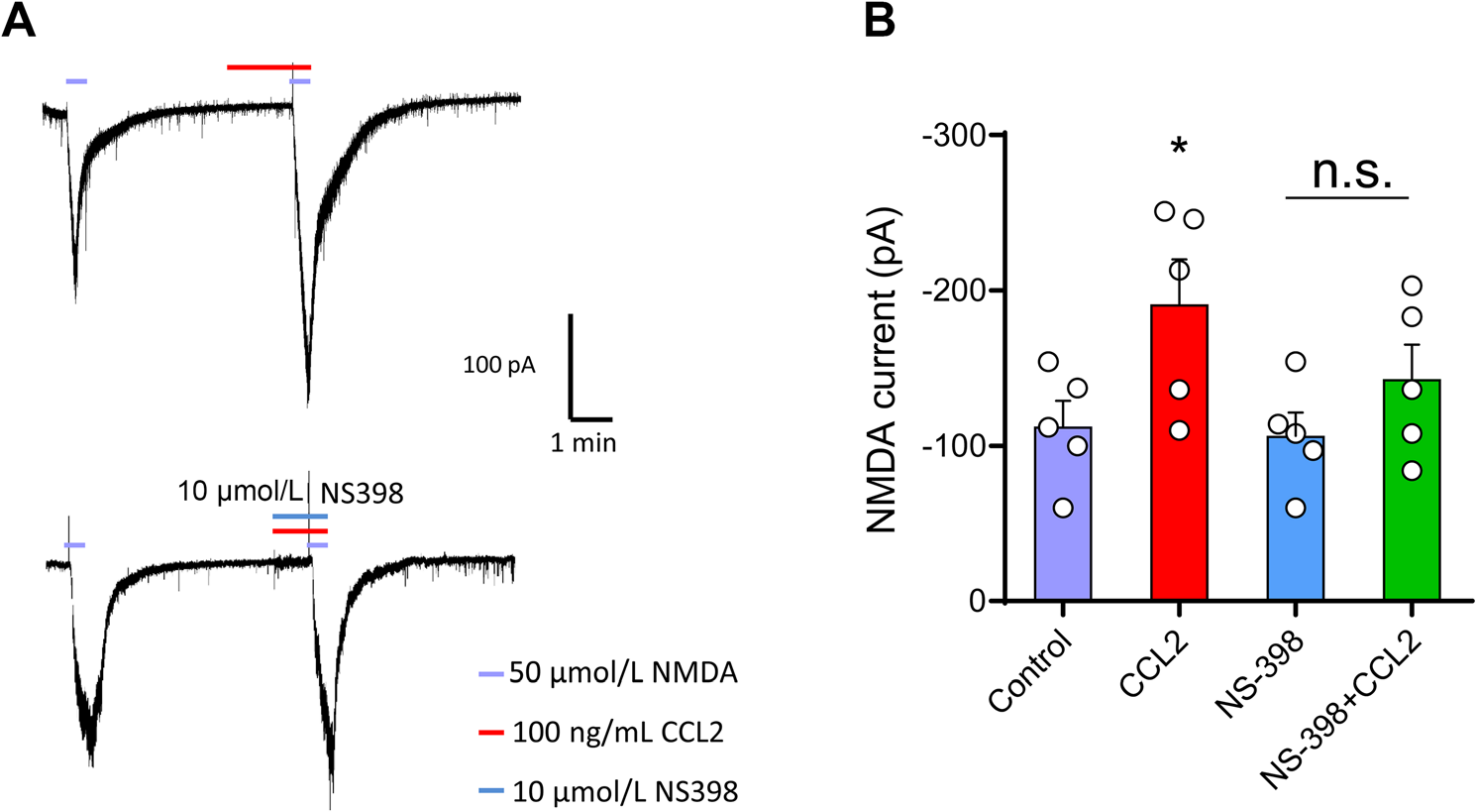
Enhancement of NMDA currents by CCL2/CCR2 *via* the Cox-2 pathway. **A, B** Perfusion of NMDA (50 μmol) at a holding potential of –40 mV induces a robust inward current which is increased by perfusion with CCL2. The enhancement of NMDA currents by CCL2 is partly blocked by NS398 (10 μm), a Cox-2 inhibitor. *P < 0.05, *n* = 5.

Cox-2 plays an important role in the inflammatory process. To determine which factors activate the CCL2/CCR2 signaling pathway and cause central sensitization, we further tested whether Cox-2 is involved in the CCL2/CCR2-mediated neuroinflammatory response. Perfusion of 50 μmol NMDA at a holding potential of –40 mV induced a robust inward current in C57Bl/6 mice. The Cox-2 inhibitor NS-398 (10 μmol) partially inhibited the enhancement of NMDA currents produced by CCL2 (Fig. 6A, B).

### The CCR2 Antagonist RS504393 and ERK Inhibitor PD98059 Abolish Spinal LTP *In Vitro*

**Fig. 7:**
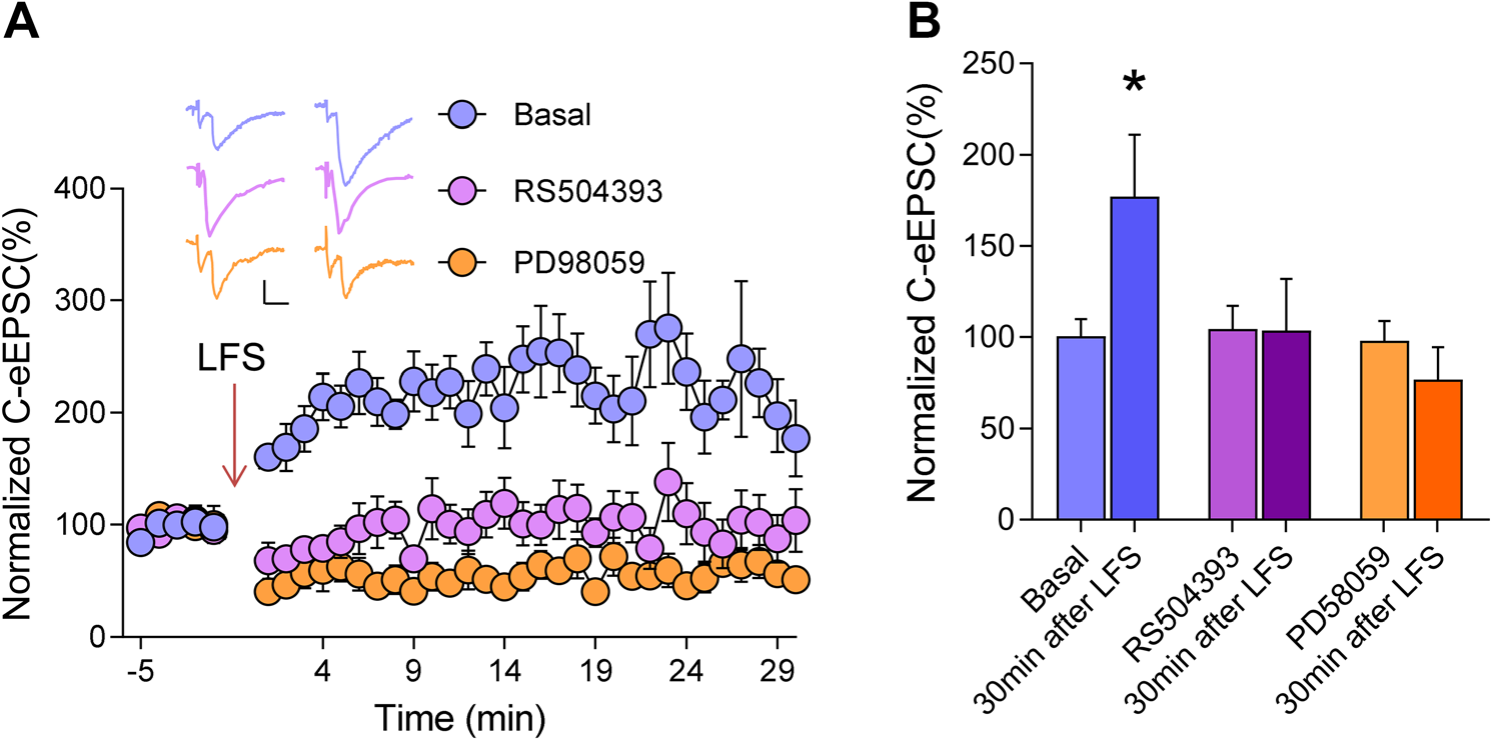
Elimination of LTP of C-fiber eEPSCs in dorsal horn neurons by RS504393 and PD98059. **A** Spinal LTP is induced by low-frequency stimulation (240 pulses at 2 Hz,) of C-fiber intensity in C57/Bl6 mice. Addition of RS504393 or PD58059 to the pipette solution largely inhibits the induction of LTP. Scale bars, 50 pA and 10 ms. **B** Quantitative summary of experiments as in A. *P < 0.05, one-way ANOVA, *n* = 5–6 neurons/group.

LTP in the spinal dorsal horn is closely associated with the generation and development of chronic pain [24]. To investigate whether LTP is induced at spinal synapses, we recorded eEPSCs in lamina IIo neurons at a holding potential of –70 mV by stimulating dorsal roots in the presence of the γ-aminobutyric acid (A) receptor antagonist gabazine (10 μmol) and the glycine receptor antagonist strychnine (1 μmol). In the lamina IIo neurons of WT mice, conditioning low-frequency stimulation (240 pulses at 2 Hz) at a holding potential of –70 mV induced LTP of monosynaptic C-fiber eEPSCs by >200% at 30 min (Fig. 7A, B). Superfusion of the CCR2 antagonist RS504393 abolished the generation of LTP, indicating an essential role of CCR2 in spinal LTP induction (Fig. 7A, B). Meanwhile, this spinal LTP was also blocked by the ERK inhibitor PD98059, suggesting the involvement of ERK activity (Fig. 7A, B).

## Discussion

**Fig. 8:**
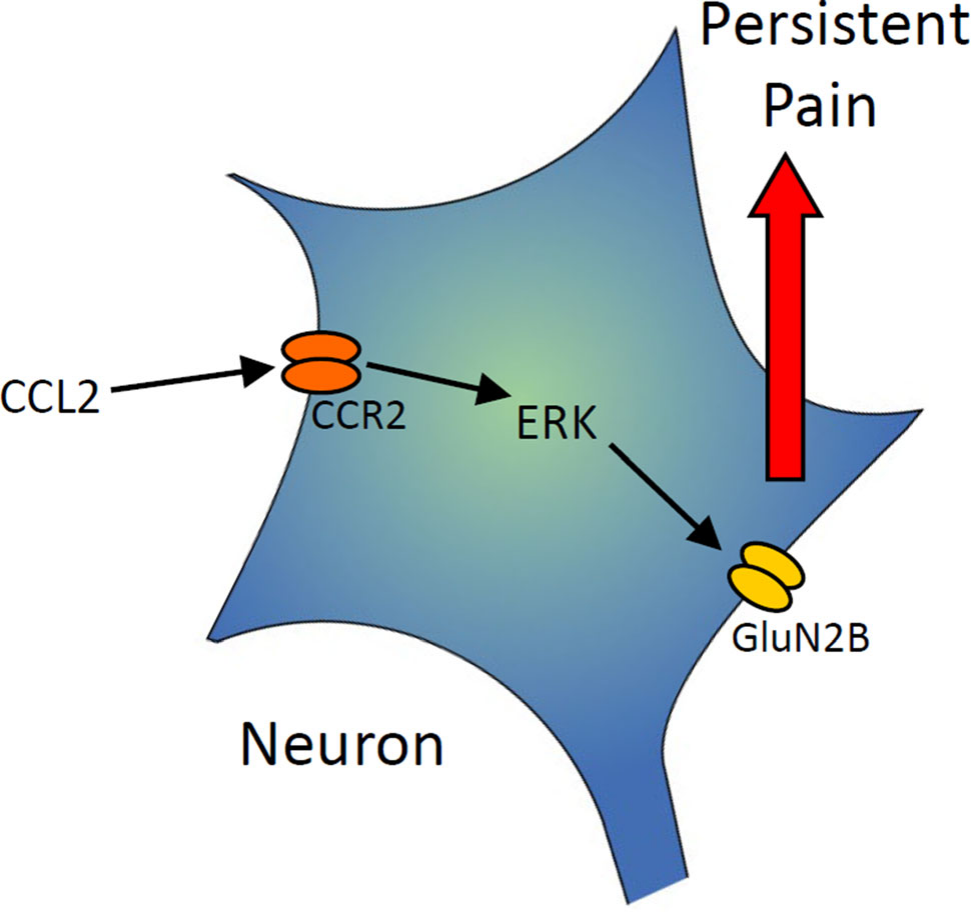
Diagram of the model. Following peripheral inflammation, directly released CCL2 acts on CCR2 on superficial dorsal horn neurons; this activates the ERK signaling cascade, leading to up-regulation of GluN2B on the cell membrane, and inducing chronic pain.

The results of the present study lead us to propose the model represented schematically in Fig. 8. Following peripheral inflammation or injury, directly released CCL2 acts on CCR2 in superficial dorsal horn neurons, leading to the activation of ERK signals and GluN2B upregulation, which in turn induces inflammatory pain.

Emerging evidence shows that, during the development of chronic pain, astrocytes regulate synaptic plasticity in the spinal dorsal horn through neuron–glia and glia–neuron interactions [25]. Excessive neuronal activity leads to the activation of glial cells and the release of cytokines and chemokines [26]. CCL2 is involved in the interaction between superficial neurons and non-neurons in the spinal dorsal horn [27]. Previous studies have shown that CCR2 is expressed in spinal microglia [18], astrocytes [22], and neurons [28]. CCR2 up-regulation has also been found in spinal microglia after partial sciatic nerve injury [18] and in astrocytes after spinal cord contusion injury [22]. Our previous reports showed that CCL2 directly regulates the synaptic plasticity of CCR2-expressing excitatory neurons in spinal lamina II, further leading to central sensitization [16]. But the specific downstream pathways of CCL2/CCR2 in superficial neurons of the dorsal horn needed to be further elucidated.

In this study, we further explored the specific downstream mechanism of action of CCL2 on CCR2 in superficial neurons of the spinal dorsal horn. First, the facilitating effect of CCL2 on NMDA-induced currents is dependent on the ERK pathway; second, CCR2 upregulation in spinal dorsal horn neurons after injury further increases the CCL2 effect and leads to central sensitization; third, CCL2 enhances NMDA currents *via* GluN2B activation; and finally, ERK is necessary for the induction of spinal LTP by CCL2–CCR2 activation.

Our previous study showed that CCL2 enhances NMDA currents only in CCR2-positive excitatory neurons that express VGLUT2 and excitatory SOM^+^ interneurons [16]. These interneurons form a nociceptive circuit by receiving inputs from C fibers that express TRPV1 (transient receptor potential cation channel subfamily V member 1) and sending the output to lamina I projection neurons that are essential for pain transmission [29–31]. Our present study extended this to show that CCL2 promoted NMDAR activity *via* phosphorylation of ERK, a marker for central sensitization [32, 33]. This provides new research ideas and further drug targets for the treatment of pain.

Studies have identified MEK1/2-ERK1/2 signaling as an important intracellular pathway that links Schwann cell mutations to the activation of pathology-associated macrophages in peripheral nerves [34]. Our results further demonstrated that the release of CCL2 in the spinal dorsal horn activated the ERK pathway through CCR2, thereby enhancing NMDAR functions.

Thacker *et al*. [35] demonstrated that intraspinal CCL2 elicits a massive microglial response in the ipsilateral dorsal horn; however, the role of endogenous CCR2 in inflammation needed to be further verified. Our study showed that CCR2 was significantly upregulated in the superficial neurons of the dorsal horn after inflammatory injury. Up-regulated CCR2 further enhanced the NMDA currents induced by CCL2. This suggests that the endogenous CCL2 release and upregulation of CCR2 in the superficial layer of the dorsal horn after inflammatory injury is an important cause of neuronal excitability. We further confirmed that CCL2-induced inflammatory pain is blocked by the ERK inhibitor PD98059. In addition, we found that intrathecal injection of PD98059 reversed the inflammatory pain caused by CFA. These results suggest that CCL2 causes inflammatory pain by activating the ERK signaling pathway. Our experiments showed that the intracellular injection of an ERK inhibitor completely blocked the enhancement of NMDA currents induced by CCL2. ERK activation in dorsal horn neurons is nociception-specific and plays an important role in the induction of central sensitization [32, 33].

The NMDAR is a well-known subtype of voltage-gated ionotropic glutamate receptor [36–38]. NMDARs specifically allow Ca^2+^ to enter postsynaptic neurons; their continuous activation leads to extracellular Ca^2+^ entry into the cell [39]. Previous studies have shown that NMDARs are abundantly expressed in the pain pathway, where they are functionally expressed not only on the soma, but also on their peripheral and central processes [40–46]. Experimental data have shown that the PKA/Fyn/GluN2B pathway triggers the enhancement of GluN2B in the dorsal horn, leading to pain hypersensitivity [47]. Spinal GluN2B plays a key role in the development and maintenance of chronic pain [48]. Our study further confirmed that NMDA currents enhanced by CCL2/CCR2 through the ERK signal pathway were mainly mediated by the GluN2B subunit, which may further lead to synaptic plasticity in the dorsal horn.

It is well known that intraplantar injection of CFA causes upregulation of Cox-2 in spinal neurons and leads to inflammatory pain [49, 50]. The Cox-2 inhibitor NS-398 partly decreased the enhancement of NMDA-induced currents. These data suggest that CCL2 is involved in the development of central sensitization and inflammatory pain, in part through the induction of nociceptive genes such as Cox2, and Cox-2 inhibitors may be potential treatments for the development of central sensitization *via* CCL2/CCR2.

Thus, pERK can serve as a marker for central sensitization in spinal dorsal horn neurons [28] and inhibition of the CCL2/CCR2 pathway may reduce central sensitization. CCL2/CCR2 enhanced GluN2B function *via* activation of the downstream target ERK, suggesting that suppression of GluN2B in the dorsal horn reduces central sensitization. Our previous research showed that RS504393, a blocker of CCR2, inhibits the maintenance of LTP [16]. In this study, we further showed that RS504393 also inhibits the induction of LTP, and this effect is achieved by activating ERK. Therefore, by inhibiting the CCL2/CCR2 pathway or inhibiting the enhancement of ERK, central sensitization can be reduced, thereby reducing the occurrence of chronic pain.
